# Attitudes Toward Obesity, Willingness to Lose Weight, and Treatment Preferences Among Overweight and Obese Saudi Adults

**DOI:** 10.7759/cureus.29228

**Published:** 2022-09-16

**Authors:** Mohammed A AlAteeq, Nuha AlHomayed, Doaa AlBuraikan, Hamzah AlFageer

**Affiliations:** 1 Family Medicine, Ministry of National Guard - Health Affairs, King Abdullah International Medical Research Center, King Saud Bin Abdulaziz University for Health Sciences, Riyadh, SAU; 2 Family Medicine and Primary Care, King Abdulaziz Medical City, Riyadh, SAU

**Keywords:** obese, overweight, obesity, behavioral medicine, physical activity, nutrition, preventive care, chronic disease

## Abstract

Background: Obesity has become a major health concern worldwide and is associated with several diseases and complications. Losing weight is an effective strategy to improve body mass index and prevent the complications of obesity. However, weight loss is dependent on the attitude of individuals toward obesity as well as their willingness to lose weight.

Aim: To explore attitudes toward obesity, willingness to lose weight, and treatment preferences among overweight and obese Saudi adults.

Methods: An analytical cross-sectional study was conducted among overweight and obese Saudis. We targeted adults aged 18 years and older, who visited family medicine clinics at King Abdulaziz Medical City for the National Guard in Riyadh, Saudi Arabia. The study was conducted from December 2020 to June 2021 using a self-administered questionnaire.

Results: Of the 403 participants, 82.5% were dissatisfied with their current body weight. Controlling chronic disease was a major motive for improving body weight (53.2%), and exercise and diet were the most preferred strategies to lose body weight. Age was a determinant in the attitude and willingness of participants to lose weight (p = 0.0001).

Conclusion: Participants in the current study reported high dissatisfaction rates about current weight and willingness to improve body weight. This should encourage healthcare providers to initiate weight status discussions and management with their overweight and obese clients.

## Introduction

Obesity has become a major health concern worldwide. It is defined as “the accumulation of adipose tissue to excess and to an extent that impairs both physical and psychosocial health and well-being” [1]. Obesity has been linked to multiple preventable comorbidities and negative health outcomes. It is a known risk factor for non-communicable diseases like hypertension, type 2 diabetes mellitus, dyslipidemia, metabolic syndrome, coronary heart disease, and certain types of cancers [2].

Obesity rates are rising globally. Between 1975 and 2014, the prevalence of obesity (body mass index (BMI) ≥ 30 kg/m2) increased from 3.2% to 10.8% in adult men and from 6.4% to 14.9% in adult women [3]. In Saudi Arabia, epidemiological studies suggest that the prevalence of obesity is increasing. A community-based national survey found a progressive increase in obesity from 22% in 1990-1993 to 36% in 2005 [4,5]. A local study discussed current trends of obesity prevalence among adults. The study predicts that the overall obesity in Saudi Arabia will rise to 41% in men and 78% in women by 2022 [6]. In a national survey conducted in 2013, including 10,735 participants, Memish et al. found a 28.7% prevalence of obesity, with higher rates among females (33.5% vs. 24.1%) [7]. This increase in obesity rates can impair people’s quality of life and adds considerably to national healthcare budgets [8].

Managing obesity as a chronic disease and setting interventional strategies aiming to reduce obesity prevalence is important. According to the health belief model, perceived personal susceptibility to disease can increase the likelihood of following the recommended actions and modify treatment-seeking behavior [9,10]. In addition, people with self-recognition of their obesity are more likely to try weight loss methods [11]. Therefore, an understanding of how people with overweight or obesity perceive their body image as well as their attitudes and willingness to lose weight is needed to provide effective and individualized strategies.

Moreover, to maximize the effectiveness of the strategies aimed at obesity control, knowledge about what overweight or obese people prefer with regard to therapeutic measures is needed. Considering patients’ preferences will enable the physician to provide an individualized and effective range of services. This study aims to explore attitudes toward obesity, willingness to lose weight, and treatment preferences among overweight and obese Saudi adults.

## Materials and methods

This work reports an analytic cross-sectional study. It was conducted at three family medicine centers at King Abdulaziz Medical City (KAMC) in Riyadh, Saudi Arabia. The study included adult patients aged 18 years and above with overweight or obesity (BMI: 25 kg/m2 and above) during their visits to family medicine clinics at the three centers, from December 2020 to June 2021. The three family medicine centers were Health Care Specialty Center (HCSC), King Abdulaziz Housing Clinics (Iskan), and the National Guard Comprehensive Specialized Clinic (NGCSC). All three centers provide primary curative and preventive health services and have both walk-in and booking appointment systems for patients to receive treatment and advice for acute and chronic medical conditions. We excluded patients under 18 years of age and patients with secondary obesity, patients diagnosed with eating disorders, pregnant women, and incompetent individuals.

The sample size was calculated based on Caterson et al., who found that 48% of the studied cohort were motivated to lose weight [12]. The calculated sample size was estimated to be 384 using a 95% confidence interval and a 5% margin of error; this was adjusted to 450 to compensate for the incomplete questionnaire. The sample size was calculated online using the OpenEpi epidemiologic calculator [13].

Data collection

Data related to participants’ demographics were obtained from their electronic medical records. This included age, gender, and BMI. BMI was defined as weight in kilograms divided by height in squared meters (kg/m2). BMI categories were defined according to WHO cut-off points, i.e., 25.0-29.9 kg/m2 for overweight and ≥30.0 kg/m2 for obesity [14]. The obesity category was further subdivided into obesity class I (BMI: 30.0-34.9 kg/m2), obesity class II (35.0-39.9 kg/m2), and obesity class III (≥40.0 kg/m2).

Data related to study objectives were collected using a self-administered questionnaire. The questionnaire was developed by the authors after a literature review and considering study outcomes. It was developed in English and then translated into Arabic. The validity of translation was ensured by forwarding and backward translation. The questionnaire was reviewed by two experts for content validation. Questionnaire piloting was done on 20 patients; the piloted group was excluded from the study sample.

The questionnaire included five sections. The first section was for sociodemographic characteristics: educational level, marital status, employment status, and if they had any chronic medical or surgical conditions. Sections two, three, and four were about perception and attitudes toward obesity, participant awareness of obesity, and willingness to lose weight, respectively. Section five was about preferences for obesity therapies: participants were asked which weight loss strategy they would prefer, including “diet only, exercise only, exercise diet, bariatric surgery, taking weight reduction medications.” Their preference was measured on a scale of “strongly preferred, preferred, neutral, not preferred, strongly not preferred.” Moreover, if dieting was their preferred method to lose weight, then they were asked about their preferred dieting plan. Participants were approached by investigators and enrolled during their routine visits to the family medicine clinic in a convenient and nonprobability sampling method.

Data analysis

Data were analyzed using Statistical Package for the Social Sciences (SPSS; IBM Corp., Armonk, NY). All statistical tests were conducted at a significance level (alpha = 0.05). Quantitative variables were reported in the form of mean and standard deviation. Qualitative variables were in the form of frequency and percentages. Chi-squared was used to compare categorical variables.

Ethical considerations

Study approval was obtained from King Abdullah International Medical Research Center (KAIMRC), Ministry of National Guard, Saudi Arabia (IRB approval number: RC20/361/R; dated: August 16, 2020). Verbal consent was obtained from participants at the time of questionnaire distribution. Privacy and confidentiality were considered and completely protected; this was used only for research purposes. Data collection sheets were coded using three‑digit serial numbers and were maintained by the co‑investigator. Participants could not be identified after the collection of the datasheets. The study was conducted according to the principles of the Declaration of Helsinki. Ethical approval was obtained from parents of individuals younger than 18 years.

## Results

The questionnaire was distributed to 450 individuals, and 403 were completed and returned with a response rate of 89.5%. The characteristics of the 403 participants are shown in Table 1. Most were females (81.1%), and more than half (62.9%) were over the age of 40. Only 1.5% of participants were overweight; the largest proportion (46.4%) had class I obesity.

**Table 1:** Characteristics of participants

Variables		Total (%)
Gender	Female	327 (81.1%)
	Male	76 (18.9%)
Age	40 or less	149 (37.1%)
	Above 40	253 (62.9%)
BMI	Overweight	6 (1.5%)
	Class 1 obesity	187 (46.4%)
	Class 2 obesity	136 (33.7%)
	Class 3 obesity	73 (18.1%)
Education	Illiterate	89 (22.1%)
	School	199 (49.4%)
	University and above	115 (28.5%)
Marital status	Married	308 (76.4%)
	Not married	95 (23.6%)
Work	Student	26 (6.5%)
	Working	88 (21.9%)
	Not working	288 (71.6%)
Comorbidity	No chronic illness	105 (26.4%)
	Single chronic disease	115 (29%)
	Multiple chronic diseases	177. (44.6%)

The perceptions and attitudes of participants toward obesity are shown in Table 2. The majority (82.5%) were dissatisfied with their current body weight, 34.1% perceived themselves as obese, and 93% considered obesity a disease. Most (78.5%) thought that obesity is a result of a single cause. The most reported motivation to lose weight was to control or cure chronic conditions. The most reported barrier to losing weight was poor determination and will. Most participants (86.6%) had tried several times to lose weight.

**Table 2:** Perception and attitude of participants toward obesity

Variables		Total (%)	
How do you perceive your weekly level of physical activity?	Inactive	186 (47.4%)	
	Active	206 (52.6)	
Are you satisfied with your current body weight?	No	330 (82.5%)	
	Yes	70 (17.5%)	
How do you see your current body size?	Very obese	71 (17.7%)	
	Obese	153 (38.1%)	
	Not obese but overweight	137 (34.1%)	
	Normal	41 (10.2%)	
Do you think you may be at risk of health problems due to your current weight?	No	134 (33.9%)	
	Yes	261 (66.1%)	
Do you think obesity is a disease?	No	28 (7%)	
	Yes	371 (93%)	
In your opinion, what is the main cause of your obesity?	No causes	14 (3.5%)	
	Single cause	314 (78.5%)	
	Multiple causes	72 (18%)	
Which of the following motivates you the most to lose weight?	Appearance/cosmetic reasons	No	300 (74.6%)
		Yes	102 (25.4%)
	To relieve the active symptoms, I am currently having due to my weight	No	286 (71.1%)
		Yes	116 (28.9%)
	To control or cure my chronic disease	No	188 (46.8%)
		Yes	214 (53.2%)
	Other	No	395 (98.3%)
		Yes	7 (1.7%)
	Not willing to lose weight	No	387 (96.3%)
		Yes	15 (3.7%)
What is the barrier that you think is preventing you from improving your weight?	No barrier, happy with my current weight	No	353 (88%)
		Yes	48 (12%)
	Poor social support	No	341 (85%)
		Yes	60 (15%)
	Lack of knowledge about weight reduction measures	No	313 (78.1%)
		Yes	88 (21.9%)
	Poor support from my doctor/dietitian	No	386 (96.3%)
		Yes	15 (3.7%)
	Poor determination and will	No	280 (69.8%)
		Yes	121 (30.2%)
	Health restrictions (e.g., joint disease, anemia, heart disease, and lung disease)	No	305 (76.1%)
		Yes	(23.9%)
Do you think that you have a strong intention and willingness to lose weight?	No	68 (17%)	
	Yes	333 (83%)	
How many times have you seriously tried to lose weight?	Zero	54 (13.4%)	
	One or two	117 (29.1%))	
	Three and more	231 (57.5%)	

Table 3 represents the methods tried by participants for weight loss. The most common method was exercising (59%), followed by avoiding or eating less junk food and fast food (50%); less common methods were having bariatric surgery (3%) and taking prescription diet injections (2%).

**Table 3:** Strategies for weight loss tried by participants

Weight loss strategy	Yes/No	N (%)
Exercising	No	165 (41%)
	Yes	237 (59%)
Drinking a lot of water	No	272 (67.7%)
	Yes	130 (32.3%)
Avoiding or eating less junk food and fast food	No	201 (50%)
	Yes	201 (50%)
Skipping meals (eating only one or two meals\day)	No	324 (80.6%)
	Yes	78 (19.4%)
Following a special diet (e.g., keto, protein, and vegetarian diet)	No	325 (80.8%)
	Yes	77 (19.2%)
Joining a weight loss program	No	376 (93.5%)
	Yes	26 (6.5%)
Taking nonprescription diet supplements	No	379 (94.3%)
	Yes	23 (5.7%)
Taking prescription diet pills (e.g., metformin/thyroxine)	No	387 (96.3%)
	Yes	15 (3.7%)
Taking prescription diet injection (e.g., Saxenda/Victoza)	No	394 (98%)
	Yes	8 (2%)
Having a bariatric surgery	No	390 (97%)
	Yes	12 (3%)

The subanalysis of participants' perception of their weight status according to the actual weight is shown in Table 4.

**Table 4:** BMI and perception of participants

BMI/perception	Very obese	Obese	Overweight	Normal
Overweight	0 (0.0%)	3 (50.0%)	1 (16.7%)	2 (33.3%)
1st stage obesity	10 (5.4%)	59 (31.7%)	91 (48.9%)	26 (14.0%)
2nd stage obesity	20 (14.7%)	69 (50.7%)	36 (26.5%)	11 (8.1%)
Morbid obesity	40 (54.8%)	22 (30.1%)	9 (12.3%)	2 (2.7%)

The preference of strategies to lose weight by participants is shown in Table 5. The preferred method was exercise and diet (83.1%), and the least preferred method was bariatric surgery (18.9%). When asked about the preferred diet to follow to lose weight, the most preferred type was decreasing unhealthy food and drinks. The least was intermittent fasting.

**Table 5:** Preference of strategies for weight loss

How much do you prefer to do the following strategies to lose weight?	Preferred	Neutral		Not preferred	
Diet only	260 (74.3%)	35 (10%)		55 (15.7%)	
Exercise only	254 (73.2%)	31 (8.9%)		62 (17.9%)	
Exercise and diet	301 (83.1%)	28 (7.7%)		33 (9.1%)	
Bariatric surgery	64 (18.9%)	17 (5%)		257 (76%)	
Take weight reduction medications	69 (20.4%)	34 (10.1%)		235 (69.5%)	
Not willing to lose weight	26 (8.2%)	31 (9.8%)		259 (82%)	
If you plan to follow a healthy diet, which type of dieting do you prefer?	Preferred		Not preferred		
Decrease intake of unhealthy food and drinks	193 (48.1%)		208 (51.9%)		
Skipping meals (eating only one or two meals\day)	53 (13.2%)		348 (86.8%)		
Following a special diet (e.g., keto, protein, and vegetarian diet)	105 (26.2%)		296 (73.8%)		
Intermittent fasting	16 (4%)		383 (96%)		

Bivariate analysis shows a significant difference between different BMI groups when asked if they think obesity jeopardizes their health, there were more affirmative answers as weight increased. In addition, the rate of dissatisfaction about current body weight increased as weight increased. Participants younger than 40 years of age were found to have more dissatisfaction rates about their current weight and were more motivated by cosmetic reasons and body shape to lose weight. They were more likely to try exercise for weight reduction relative to older participants (Table 6).

**Table 6:** Significant associations with weight status, age, and gender

Variables		Overweight	1st stage obesity	2nd stage obesity	Morbid obesity	P-value
Are you satisfied with your current body weight?	Yes	2 (33.3%)	41 (22.2%)	22 (16.2%)	4 (5.6%)	0.011
	No	4 (66.7%)	144 (77.8%)	114 (83.8%)	68 (94.4%)	
Do you think you may be at risk of health problems due to your current weight?	Yes	3 (50%)	94 (51.6%)	99 (73.9%)	64 (88.9%)	0.000
	No	3 (50%)	88 (48.4%)	35 (26.1%)	8 (11.1%)	
Variables		Age 40 or less		Age above 40		P-value
Are you satisfied with your current body weight?	Yes	10 (6.8%)		60 (23.8%)		0.000
	No	137 (93.2%)		193 (76.2%)		
Do appearance/cosmetic reasons motivate you the most to lose weight?	Yes	61 (41.2%)		41 (16.2%)		0.000
	No	87 (58.8%)		212 (83.8%)		
Have you tried exercising before to lose weight?	Yes	104 (70.3%)		132 (52.2%)		0.000
	No	44 (29.7%)		121 (47.8%)		
Do health restrictions (e.g., joint disease, anemia, heart disease, and lung disease) prevent you from improving your weight?	Yes	13 (8.8%)		82 (32.5%)		0.000
	No	135 (91.2%)		170 (67.5%)		
Variables		Male		Female		P-value
Have you tried avoiding or eating less junk food and fast food before to lose weight?	Yes	25 (32.9%)		176 (54%)		0.001
	No	51 (67.1%)		150 (46%)		

Patients with chronic medical conditions, either single or multiple, consider their health restrictions (e.g., joint disease, anemia, heart disease, and lung disease) a barrier to improving their weight. Likewise, patients with multiple comorbid conditions are motivated to lose weight more by the idea that losing weight will relieve the active symptoms they currently have. In addition, educational level was associated with more awareness about the health risks of obesity (Table 7).

**Table 7:** Significant associations between educational level and morbidities

Variable		Illiterate	School	University and above	P-value
Do you think you may be at risk of health problems due to your current weight?	Yes	45 (50.6%)	136 (69.4%)	80 (72.7%)	0.002
	No	44 (49.4%)	60 (30.6%)	30 (27.3%)	
Variables		No chronic illness	Single chronic disease	Multiple comorbid conditions	P-value
In my opinion, the main cause of my obesity is the Medications I am currently taking.	Yes	1 (1%)	2 (1.8%)	14 (7.9%)	0.006
	No	103 (99%)	112 (98.2%)	163 (92.1%)	
To relieve the active symptoms I am currently having due to my weight motivates me the most to lose weight.	Yes	17 (16.3%)	27 (23.5%)	72 (40.7%)	0.000
	No	87 (83.7%)	88 (76.5%)	105 (59.3%)	
Health restriction is preventing me from improving my weight (e.g., joint disease, anemia, heart disease, and lung disease).	Yes	5 (4.8%)	21 (18.4%)	70 (39.5%)	0.000
	No	99 (95.2%)	93 (81.6%)	107 (60.5%)	

## Discussion

Our data indicate that most overweight or obese people are dissatisfied with their current weight. More than half of them think that they are either obese or very obese. A large majority knew that obesity is a disease. Most reported that obesity is caused by a single cause. In a similar study done in Lithuania, almost two-thirds of 198 people with obesity were either unhappy or very unhappy with their current weight [15]. This attitude about obesity and the perception of it as a disease is encouraging because it forms a good basis for healthcare providers to initiate obesity management.

Saudi data from the ACTION International Observation (ACTION-IO) study by Alfadda et al. included 1,000 Saudis with obesity: 87% agreed that obesity has an enormous impact on health, and 68% considered it to be a chronic disease [16]. A similar rate was reported among American people with obesity [17].

Another local study evaluated Saudi females attending fitness centers and found that less than half of them underestimated their perceived body shape (40%). The majority (87%) were dissatisfied with their body shape, but, of these, 68% had normal weight [18]. This attitude, the dissatisfaction of people with normal weight with their body shape, is undesirable and may lead to unhealthy behavior.

The dissatisfaction rate was higher among people aged 40 or less, and the difference was statistically significant. This is understandable since younger people are expected to be more concerned about self-image and looks. As we might expect, the rate of dissatisfaction about current weight and perceived health risk of obesity both move proportionately to increased obesity levels. In a systematic review, aging was associated with decreased concern about body weight and less overweight or obesity self-perception [19]. This is important and should be considered when discussing obesity management with patients to encourage them to start weight management at an early age.

We found no gender difference in terms of dissatisfaction with current weight. This may be attributed to a nonmatching sample size of males with females (76 vs. 327). However, one study by Tsai et al. in the United States found that men are less accurate in their weight perceptions and weight dissatisfaction, i.e., their perception is less consistent with the actual body weight compared to women [20]. Similar findings were also reported among a Mediterranean adult population [21]. In a small local study, 18.4% of overweight or obese young males reported their weight as appropriate [22].

A previous Saudi study assessed the attitudes of adults toward obesity and showed that age, education level, and BMI were determinants for attitudes toward obesity [23] similar to our study. Age also significantly affected lifestyle-changing behavior, with younger people being more likely to change their lifestyle, as reported by Zelenyte et al. [15]. The willingness to lose weight was reported to be affected by BMI and gender, i.e., obese participants, especially men, showed a willingness to reduce weight more than men and women who are overweight [24]. A similar study in the United States found a significant difference between different ethnic groups in terms of self-recognition of obesity and views of obesity as a health problem, with whites being more likely to self-report obesity compared to Hispanics and African-Americans [11].

Of note, one-third of the participants did not think that they are at risk of health problems due to their current weight. This was more common among participants with low educational levels; it was statistically significant. This is worrying and patient education is needed to correct this misconception.

The vast majority of participants in this study perceived their intention and willingness to lose weight as strong. In comparison, a local study found that almost half of the investigated people with obesity were motivated to lose weight [13]. For comparison to international figures, we note the ACTION-IO study of 14,502 people with obesity across 11 countries; here, 48% were motivated to lose weight [12].

The major motive for our participants to lose weight was to control their chronic disease followed by relieving active symptoms. The main motives in other studies were different and included concerns about overall health, a desire to improve their look, to be more confident, to improve self‑esteem, and to be more fit [16,25]. The difference here may be related to the type of study, which was community-based for the previous studies while this study analyzed visitors to family medicine clinics. About 63% were over the age of 40, and more than 70% of them have one or more chronic diseases. Considering age again, older participants were motivated by body shape and cosmetic appearance less than younger ones.

Most participants did not agree on the listed possible barriers to weight loss. This means no reported perceived barrier by the majority. The most frequent barrier (30% of participants) was poor determination and will. This is similar to the previous answer about perceived intention and willingness to lose weight where about 20% reported having weak intention and willingness. Another significant finding seen here is that almost 40% of participants with multiple chronic conditions considered health restrictions like joint disease, anemia, heart disease, and lung disease as a barrier to not losing weight. This must be considered when educating patients about lifestyle modifications and physical activities; these people need more appropriate types of exercise and control of disabling conditions.

In another local study, and in contrast to our findings, a lack of family support, unhealthy eating during social gatherings, and declining motivations were major barriers to weight loss [25]. Interestingly, a local study found that genetic factors were barriers to weight loss for 39% of participants [16]. Obesity is a multifactorial condition, and genetics certainly play a role; however, this has nothing to do with the ability and possibility to lose weight [26].

One-third of participants reported one or two serious attempts to lose weight, and about 60% had tried three or more attempts similar to findings by local and international studies [11,16,18]. In a local study done on overweight and obese women attending a diet clinic in Riyadh, the current visit to the diet clinic was the first trial to lose weight for 19.8% of participants, and 33.4% reported more than four attempts [27]. Lower figures were reported in an American study [18]. In a meta-analysis of 72 studies of 1,184,942 people from different Asian, European, and American countries, 42% reported at least one trial of weight reduction [28].

To succeed, it is particularly important for overweight or obese people to recognize their responsibility for weight loss and reaching a healthy weight. Here, we did not directly ask participants about their responsibility for obesity management; rather, a prior study found that 82% of respondents agreed that weight loss is their responsibility [17]. Healthcare workers (HCWs) play a key role in the initiation of obesity management and support people with obesity to lose weight. In a local study, 85% felt positive and 54% felt hopeful after a weight management discussion with HCWs [16]. This implies that more education and training for HCWs are needed for them to be confident and professional in discussing weight management with their patients.

The most preferred strategies to lose weight among our participants were exercise and diet (83.1%), followed by diet only (74.3%) and exercise only (73.2%). Similar findings were reported in two other studies [20,27]. This indicates that the major focus of participants was on exercise and diet. The major preferred plan related to diet control was to reduce the intake of unhealthy food and drinks. Food intake is perceived as a major cause of obesity. One study reported that food intake habits were a personalized bodyweight-determining factor with a strong impact on Saudi weight management [29]. This can be attributed to the fact that junk food consumption in Saudi Arabia is increasingly common [7].

Interestingly, weight reduction medication was the preferred way of weight loss by only 20% of participants. This contradicts the assumption that people with obesity and overweight may prefer easy and quick weight-loss strategies.

Limitations

The non-probability convenient sampling way is considered a limitation. The number of males in this study did not match the number of females and there was no control group. Another limitation is that the study was not community-based, and this may limit the generalizability of the findings. Having no control group may be considered a limitation.

## Conclusions

The current study reported high dissatisfaction rates about current weight and willingness to improve body weight among overweight and obese people. However, their attitude and willingness were affected by certain modifiable demographics such as education level.
